# Spectrum of Epithelial-Mesenchymal Transition Phenotypes in Circulating Tumour Cells from Early Breast Cancer Patients

**DOI:** 10.3390/cancers11010059

**Published:** 2019-01-09

**Authors:** Aleksandra Markiewicz, Justyna Topa, Anna Nagel, Jaroslaw Skokowski, Barbara Seroczynska, Tomasz Stokowy, Marzena Welnicka-Jaskiewicz, Anna J. Zaczek

**Affiliations:** 1 Department of Medical Biotechnology, Intercollegiate Faculty of Biotechnology of the University of Gdansk and Medical University of Gdansk, 80-211 Gdańsk, Poland; justyna.topa@gumed.edu.pl (J.T.); anna.nagel@biotech.ug.edu.pl (A.N.); 2 Department of Surgical Oncology, Medical University of Gdansk, 80-210 Gdańsk, Poland; jskokowski@gumed.edu.pl; 3 Department of Medical Laboratory Diagnostics-Biobank, Medical University of Gdansk, 80-210 Gdańsk, Poland; bastrzel@gumed.edu.pl; 4 Biobanking and Biomolecular Resources Research Infrastructure (BBMRI.PL), 80-210 Gdansk, Poland; 5 Department of Clinical Science, University of Bergen, 5007 Bergen, Norway; tomasz.stokowy@k2.uib.no; 6 Department of Oncology and Radiotherapy, Medical University of Gdansk, 80-210 Gdańsk, Poland; mwelj@gumed.edu.pl

**Keywords:** circulating tumour cells, epithelial-mesenchymal transition, cancer stem cell markers, breast cancer, prognostic factor

## Abstract

Circulating tumour cells (CTCs) can provide valuable prognostic information in a number of epithelial cancers. However, their detection is hampered due to their molecular heterogeneity, which can be induced by the epithelial-mesenchymal transition (EMT) process. Therefore, current knowledge about CTCs from clinical samples is often limited due to an inability to isolate wide spectrum of CTCs phenotypes. In the current work, we aimed at isolation and molecular characterization of CTCs with different EMT status in order to establish their clinical significance in early breast cancer patients. We have obtained CTCs-enriched blood fraction from 83 breast cancer patients in which we have tested the expression of epithelial, mesenchymal and general breast cancer CTCs markers (*MGB1/HER2/CK19/CDH1/CDH2/VIM/PLS3*), cancer stem cell markers (*CD44*, *NANOG*, *ALDH1*, *OCT-4*, *CD133*) and cluster formation gene (plakoglobin). We have shown that in the CTCs-positive patients, epithelial, epithelial-mesenchymal and mesenchymal CTCs markers were detected at a similar rate (in 28%, 24% and 24%, respectively). Mesenchymal CTCs were characterized by the most aggressive phenotype (significantly higher expression of *CXCR4*, *uPAR*, *CD44*, *NANOG*, *p* < 0.05 for all), presence of lymph node metastases (*p* = *0.043*), larger tumour size (*p = 0.023*) and 7.33 higher risk of death in the multivariate analysis (95% CI 1.06–50.41, *p = 0.04*). Epithelial-mesenchymal subtype, believed to correspond to highly plastic and aggressive state, did not show significant impact on survival. Gene expression profile of samples with epithelial-mesenchymal CTCs group resembled pure epithelial or pure mesenchymal phenotypes, possibly underlining degree of EMT activation in particular patient’s sample. Molecular profiling of CTCs EMT phenotype provides more detailed and clinically informative results, proving the role of EMT in malignant cancer progression in early breast cancer.

## 1. Introduction

Circulating tumour cells (CTCs) are detected in peripheral blood and are prognostic in patients with different solid tumours including breast [1,2], colorectal [3,4], lung [5], prostate [6] cancer and hepatocellular carcinoma [7]. A number of methods for CTCs detection and isolation have been described, enriching CTCs based on their biological or physical features, like size, density, deformability, markers expression or secretion of proteins [7,8,9,10,11,12,13]. The main limitation of the current methods is the inability to isolate different populations of CTCs due to their phenotypic differences [14,15,16]. The dynamically developing field of CTCs detection and characterization revealed that CTCs exhibit inter- and intra-patient heterogeneity, also in terms of markers that are used to define CTCs presence [14,17]. This heterogeneity can impact ability to detect CTCs, which is especially important in early stage disease, when CTCs are rare (1–2 cells/7.5 mL of blood) and could be lost due to lack of specific marker expression [1,18,19]. 

The best known phenomenon, which is involved in introducing such heterogeneity is epithelial-mesenchymal transition (EMT), a physiological process hijacked by cancer cells. It allows cancer cells to curtail epithelial features, which restrict polarity and promotes mesenchymal phenotype, characterized by a higher migratory and invasive potential as well as increased level of stem cell markers [20,21,22,23]. Activation of EMT in cancer has important functional consequences, as mesenchymal cancer cells were shown to be more invasive and therapy resistant. Upon disease progression fraction of mesenchymal CTCs increases [17] and is linked with the development of distant metastases [24]. 

EMT was commonly viewed as a process causing complete transformation from the epithelial to the mesenchymal state. However, as a result of partial EMT, CTCs may obtain a broad spectrum of EMT phenotypes and possesses different features, translating to their plasticity [17,21,25,26,27,28]. It is speculated, that tumour cells in partial EMT state would be associated with a worse outcome than cells, which have undergone complete EMT, as a result of their increased adaptability, allowing them to convert to epithelial or mesenchymal state in response to environmental factors [29,30]. Nevertheless, data from clinical samples are still needed to prove the increased malignancy of CTCs with concurrent epithelial and mesenchymal features, particularly in the early cancer stages, where therapeutic intervention is the most efficient. It is therefore important, to evaluate not only the presence of CTCs but also their phenotype. The difficulty lies in the selection of appropriate CTCs markers, as commonly used epithelial markers such as epithelial cellular adhesion molecule (EpCAM), cytokeratin-19 (CK19) and E-cadherin (encoded by *CDH1* gene), can be downregulated during EMT, thus, their utility to detect entire population of CTCs is being questioned [31,32,33,34]. CTCs undergoing EMT acquire mesenchymal morphology, which is associated with full or partial CK19 replacement by vimentin (VIM) [35]. Although VIM may be expressed in carcinoma cells, it is also present in blood cells, what limits its usage as CTCs marker alone [12], underlines the need to monitor leukocyte contamination and stresses the urge to identify novel, specific mesenchymal CTCs markers. Plastin 3 (PLS3) was recently described as a potential marker of breast and colorectal cancer CTCs, including those after EMT [36,37]. Its overexpression enables tumour cells to form metastases, avoid anoikis and survive during therapy [37]. In human cancer cell lines high *PLS3* expression correlates with mesenchymal, stemness and metastatic gene expression signature, which suggests that *PLS3* may also be treated as a marker of aggressiveness and metastatic potential [37]. In case of CTCs-enriched blood samples *PLS3* could constitute a more specific mesenchymal marker of cancer cells. Yet another mesenchymal marker used for CTCs detection is N-cadherin (encoded by *CDH2* gene), which increased expression is accompanied by downregulation or re-localization of epithelial E-cadherin (so called cadherin switch) [38]. As a result, cells after EMT form weaker adherent junctions between adjacent cells, what increases their motility [39]. However, E-cadherin should not be seen only as a factor preventing tumour spread. It is also responsible for tumour cells clusters formation, which are more efficient at seeding metastases than single cells [40]. Newly described marker of breast cancer cells cluster formation–plakoglobin (encoded by *JUP* gene)—was also related to higher number of lung metastases in mouse models and worse survival of patients with high expression of plakoglobin in primary tumours [40]. Tan et al., described that breast cancer cell lines with high EMT score showed significantly higher VIM and N-cadherin proteins expression than cell lines with epithelial features [30]. Consequently, cell lines with low EMT score displayed higher positive staining for CK19 and E-cadherin. What is important, cell lines with intermediate EMT status exhibited medial expression level of CK19 and E-cadherin proteins, which shows that these markers are suitable for describing EMT states with highest plasticity and in consequence with the greatest metastatic potential. Apart from epithelial and mesenchymal CTCs markers, in the current study we have chosen a general breast cancer transcripts–mammaglobin A (encoded by *MGB1*) and *HER2* for the detection of CTCs. *MGB1* is exclusively expressed in cells originating from mammary gland and it is overexpressed in some breast cancer cell lines [41]. *HER2* is overexpressed in 20% of breast cancers (but overall is expressed in 60% of breast cancers above the healthy breast tissue level [42], thus not restricted only to HER2-positive tumours) and together with *MGB1* show in a numbers of studies clinical validity in detection of breast cancer CTCs [31,43,44,45,46,47,48,49,50,51]. Summary of all genes tested is presented in Table S1.

In the current work we hypothesized that using multiple markers described above will allow us to capture a wide range of CTCs using general breast cancer markers—*MGB1*, *HER2* as well as epithelial (*CK19*, *CDH1*) and mesenchymal markers (*VIM*, *CDH2*, *PLS3*) for CTCs’ EMT phenotype determination. Such CTCs were further characterized in terms of gene expression profile linked with malignant features, such as cluster formation markers, invasion and metastasis related genes, stem cell markers and their clinical significance was assessed. 

## 2. Results

**Figure 1:**
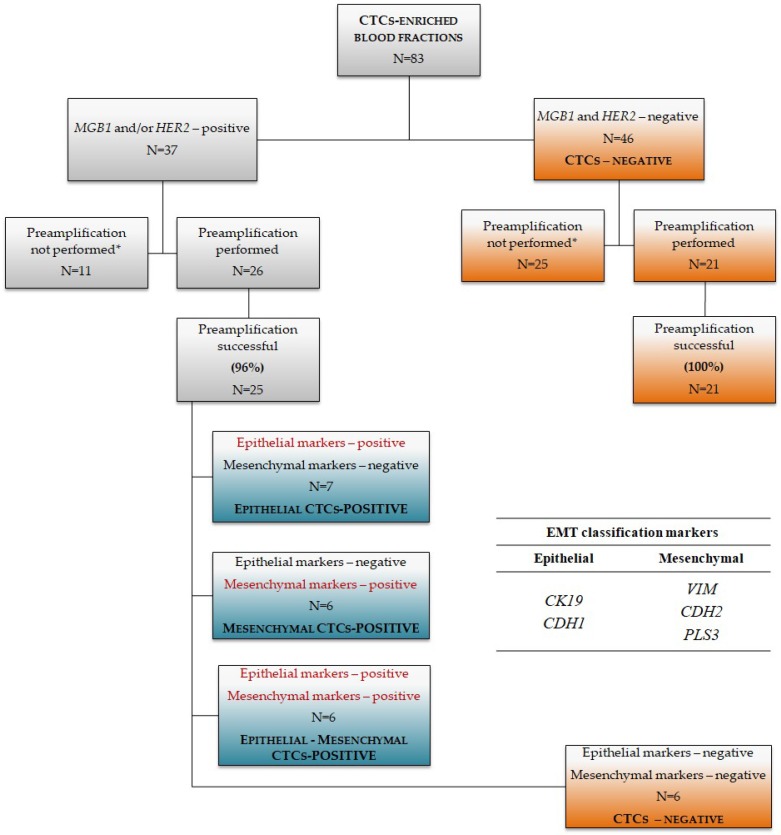
Description of samples included and excluded from the analysis in the study. Criteria used for CTCs-positivity determination were the expression of *MGB1* and/or *HER2* and division into EMT classes was based on the expression of *CK19*, *VIM*, *CDH1*, *CDH2*, *PLS3* (*—preamplification not performed due to low amount or insufficient quality of the starting material).

Thirty seven out of eighty three (45%) tested CTCs-enriched blood fractions (further referred to as CTCs-EBF) were positive for general breast cancer markers *MGB1* and *HER2*, and overall 19/46 (41%) preamplified samples were CTCs-positive (expressing *MGB1* and/or *HER2* and at least one epithelial or mesenchymal marker; Figure 1). In the *MGB1* and/or *HER2* -positive group preamplification was successful for 25/26 (96%) of samples, in the *MGB1* and *HER2*-negative group (no *MGB1* or *HER2* expression detected above the threshold level) all samples (21/21) were preamplified successfully (Figure 1). Results of further gene expression analysis of the *MGB1* and/or *HER2-*positive preamplified samples allowed for classification into three EMT classes—epithelial (*CK19-* and/or *CDH1*-positive), mesenchymal (*VIM-* and/or *CDH2-* and/or *PLS3*-positive) and epithelial-mesenchymal (biphenotypic) group in which both epithelial (*CK19* and/or *CDH1*) and mesenchymal markers (*VIM* and/or *CDH2* and/or *PLS3*) were co-expressed. Seven out of twenty five (28%) *MGB1-* and/or *HER2*-positive samples had epithelial phenotype, 6/25 (24%) had mesenchymal phenotype and 6/25 (24%) showed epithelial-mesenchymal features (Figure 1). Six samples showed no expression of any epithelial or mesenchymal CTCs markers, thus they were classified as CTCs negative. In order to classify a sample as positive for a given epithelial or mesenchymal marker expression of this markers had to be higher than in healthy control group.

None of the CTCs-EBF obtained from healthy donors had detected expression of *MGB1*, *HER2*, *CK19* and *uPAR* (Figure S1, Table S2). Expression of *VIM*, *CDH1*, *CDH2*, *PLS3*, *CXCR4*, *CD44* and *NANOG* have been found in healthy controls and the maximal expression levels of these genes were the threshold beyond which CTCs-EBF of breast cancer patients were considered positive (Table S2, Figure S1). Interestingly, CTCs-negative breast cancer samples had increased level of *CDH2* in comparison to CTCs-positive samples (average expression 31.31 vs. 19.53, respectively; *p* = 0.020; Figure S1). Similar trend was observed for *CDH1*, but their prognostic significance is difficult to reliably assess due to the low number of marker-positive samples (only two *CDH2*-positive samples were found in CTCs-negative group; Figures S1 and S2). 

### 2.1. Gene Expression Analysis in CTCs with Different EMT Status

**Figure 2:**
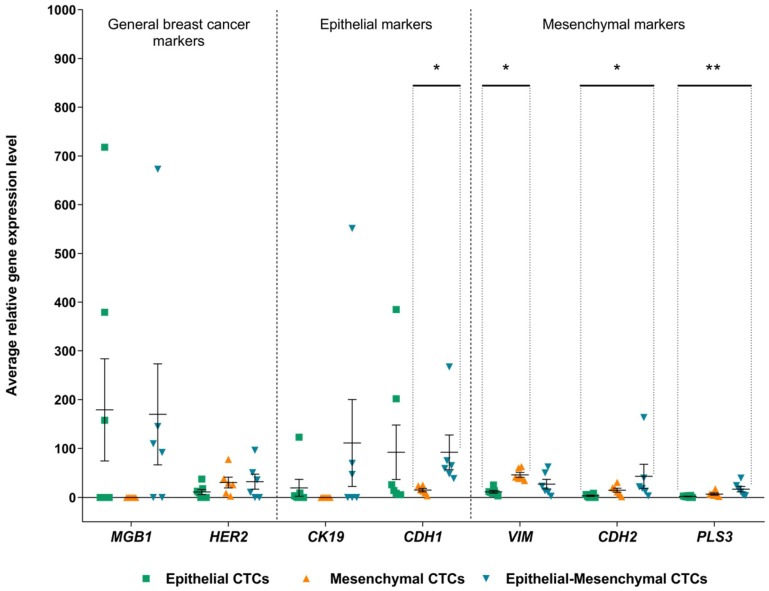
Relative expression level of genes used for CTCs-positivity determination and distribution into classes. Gene expression levels were scaled to the sample with the lowest expression of the gene of interest. Bars depict standard error and horizontal line shows mean. Statistically significant differences are marked (* *p* < 0.05, ** *p* < 0.01).

*CK19*, *CDH1*, *VIM*, *CDH2*, *PLS3* were used to classify CTCs-positive samples into EMT classes, thus it was expected to see differences in their expression among the groups (especially between pure epithelial and pure mesenchymal samples). However, it was unknown how epithelial-mesenchymal CTCs will differ from the epithelial and mesenchymal groups. In order to determine the variation, we have compared the level of general breast cancer markers (*MGB1*, *HER2*) and genes used for CTCs-EBF classification into EMT phenotypes (*CK19*, *CDH1*, *VIM*, *CDH2*, *PLS3*). There were significant differences in expression of mesenchymal genes—*VIM, CDH2* and *PLS3* (Figure 2). As expected, average *VIM* expression was higher in mesenchymal compared to epithelial CTCs-positive samples (11.59 vs. 46.23, *p* = 0.01), whereas *CDH2* and *PLS3* were increased in epithelial-mesenchymal compared to epithelial CTCs-positive samples (*CDH2* 43.26 vs. 3.31, *p* = 0.012; *PLS3* 16.86 vs. 2.01, *p =* 0.005, respectively).

*MGB1* (which was not used for classification into EMT phenotypes) showed the lowest expression in samples with mesenchymal phenotype, which would suggest *MGB1* downregulation during EMT, but the difference was not statistically significant (*p =* 0.097). Similarly *CDH1* had reduced expression in mesenchymal CTCs, the difference was statistically significant when compared with epithelial-mesenchymal CTCs (*p =* 0.036) (Figure 2). Status of each CTCs-positive sample for tested epithelial and mesenchymal genes is depicted in Figure S3.

**Figure 3:**
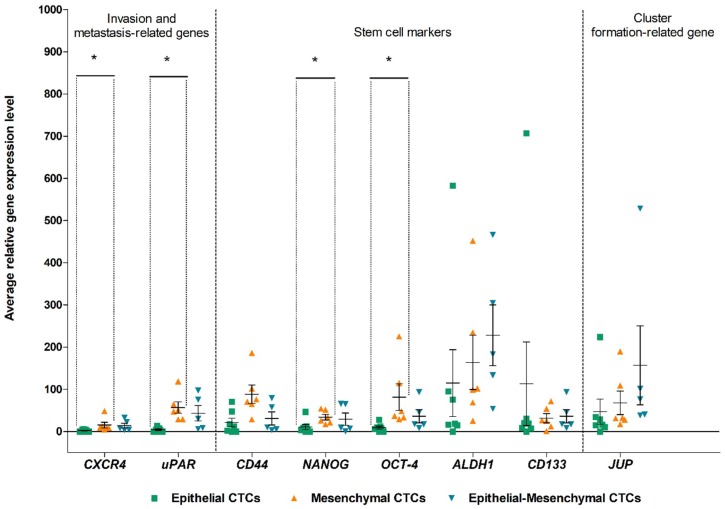
Comparison of invasion-related (*CXCR4*, *uPAR*), stem cell (*CD44*, *NANOG*, *OCT-4, ALDH1*, CD133) and clusters formation (*JUP*) markers expression levels between epithelial, mesenchymal and epithelial-mesenchymal CTCs-positive samples. Gene expression levels were scaled to the sample with the lowest expression of the gene of interest. Bars depict standard error and horizontal line shows mean. Statistically significant differences are marked (* *p* < 0.05).

Next, we looked at the expression of CSCs markers (*CD44*, *NANOG*, *OCT*-4, *ALDH1, CD133*), invasion-related genes (*CXCR4*, *uPAR*) and a marker of clusters formation (*JUP*) in different EMT classes of CTCs-positive samples (Figure 3). With the appearance of mesenchymal properties *CXCR4*, *uPAR*, *NANOG* and *OCT-4* increased their expression. The differences were the most significant when comparing epithelial and mesenchymal phenotypes (average *CXCR4* expression respectively in epithelial and mesenchymal phenotypes was 3.32 vs. 15.32, *p* = 0.035; for *uPAR* 7.95 vs. 56.57, *p* = 0.018; for *NANOG* 6.33 vs. 33.73, *p* = 0.029; for *OCT*-*4* 15.02 vs. 81.30, *p* = 0.033).

**Figure 4:**
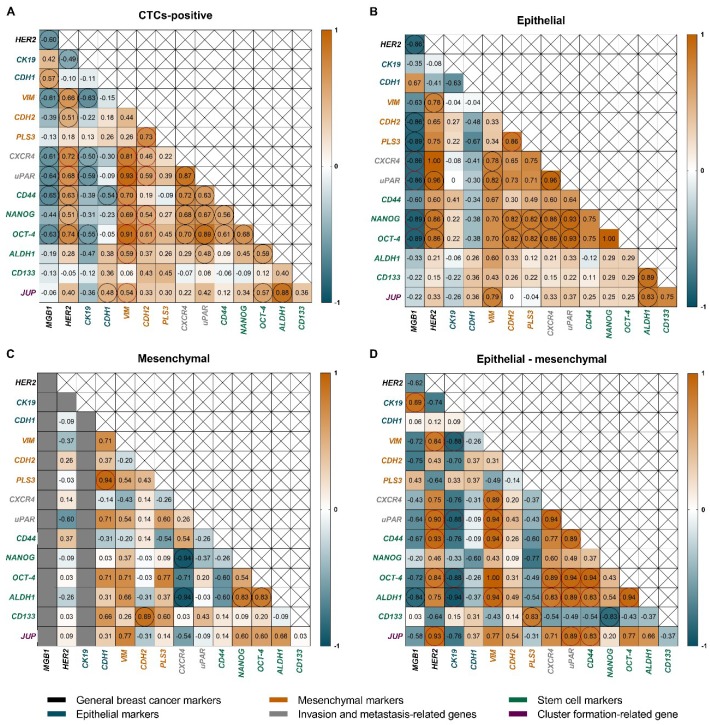
Spearman’s correlations coefficients (numbers in individual boxes) between tested genes expression level in the whole CTCs-positive group (**A**); as well as CTCs-positive samples divided into EMT classes-epithelial (**B**); mesenchymal (**C**) or epithelial-mesenchymal (**D**). Statistically significant (*p* < 0.05) results are marked in circles, results discussed in text are additionally marked in red. Strength of correlation: ρ_S_ = 0.9–1.0—perfect correlation, 0.8–0.9—very strong correlation, 0.6–0.8—strong correlation, 0.4–0.6—moderate correlation, 0.2–0.4—weak correlation, <0.2—very weak correlation.

To show connections between genes in different CTCs classes, Spearman’s rank order tests were performed. Mesenchymal markers (*VIM*, *CDH2*) strongly correlated with invasion-related (*CXCR4*, *uPAR*), stemness (*NANOG*, *OCT-4*, *ALDH1*) and cluster formation (*JUP*) genes (Figure 4A). *CXCR4* and *uPAR* also positively correlated with *HER2* (ρ_S_ = 0.72, *p* = 0.0005 and ρ_S_ = 0.68, *p* = 0.001, respectively), as they were shown to be involved in *HER2* signaling and could be jointly amplified [52,53]. The strength of the observed correlation increased when samples were analyzed separately, as distinct EMT phenotypes.

In the epithelial group, looking at the genes with the highest expression (presented on Figure 2)—*MGB1* was negatively correlated with metastasis-related (*CXCR4*, *uPAR;* ρS = −0.86, *p* = 0.01 for both) and stem cell genes (*OCT-4* and *NANOG* ρ_S_ = −0.89, *p* = 0.006; for both) (Figure 4B), what could indicate decreased malignancy of epithelial CTCs. What is interesting, in the epithelial group expression of *PLS3* correlated with mesenchymal cadherin—*CDH2* (ρ_S_ = 0.86, *p* = 0.01), but not with epithelial cadherin—*CDH1* (ρ_S_ = −0.67, *p* = 0.10) (Figure 4B), whereas opposite situation was noted in the mesenchymal CTCs-positive group—*PLS3* strongly correlated with *CDH1* (ρ_S_ = 0.94, *p* = 0.005), but not with *CDH2* (ρ_S_ = 0.43, *p* = 0.396) (Figure 4C). Switch in *PLS3* correlations with different cadherins might reveal its role in EMT phenotypic plasticity of CTCs. In epithelial-mesenchymal group the widest spectrum of correlations between genes expression was visible (Figure 4D). *CK19* was positively correlated with *MGB1* (ρ_S_ = 0.89, *p* = 0.02), but also with reduction of mesenchymal *VIM (*ρ_S_ = −0.88, *p = 0.02*)), metastasis marker (*uPAR;* ρ_S_ = −0.88, *p* = 0.02) and stem cell markers (*OCT-4* ρ_S_ = −0.88, *p* = 0.02, *ALDH1*; ρ_S_ = −0.94, *p* = 0.005), implying that detection of epithelial transcripts in the biphenotypic group is linked with less aggressive characteristics. On the other hand, in the epithelial-mesenchymal group we observed correlations between mesenchymal CTCs markers (*VIM*, *HER2*) and genes related to increased malignancy (*CXCR4*, *uPAR*, *CD44*, *OCT-4*, *ALDH1*), or cluster formation marker (*JUP*), what argues for the role of the mesenchymal markers in determination of aggressive phenotype.

Estimation of leukocyte contamination with the expression of *CD45* (encoded by *PTPRC* gene) revealed that *CD45* level is similar in healthy controls and cancer patients (26.80 vs. 25.25, *p =* 0.393—Figure S4). Additionally, we have correlated the expression of *CD45* with the level of mesenchymal markers used for CTCs calling specifically *VIM*, *PLS3*, *CDH2* (Table S3). We have divided CTC-positive samples into individual marker positive (*VIM+* or *PLS3+* or *CDH2+*) and marker negative (*VIM-* or *PLS3-* or *CDH2-*) based on the threshold determined from the analysis of healthy controls (if expression of a marker in the patient’s sample was greater than the highest expression of a marker in healthy controls, sample was considered marker-positive). We have found that for *PLS3* and *CDH2* there was no correlation with *CD45* in any of the groups, and for *VIM* correlation was observed, but only in the *VIM-*negative subgroup ρ_S_ = 0.78, *p* = 0.004; Table S3). In the *VIM*-positive group (*VIM* expression greater than in healthy controls) there was no correlation with *CD45* expression (ρ_S_ = 0.02, *p* = 0.955). When CTCs samples were divided to EMT classes—no correlation was observed between *CD45* and *VIM* in the mesenchymal group (ρ_S_ = −0.31, *p* = 0.544), as in this class all the samples had *VIM* level higher than in the healthy controls. Presence of the correlation in the epithelial-mesenchymal group can be explained by the mixed status of the group, in which 4/6 samples had *VIM* expression below the cut-off in healthy control (but their mesenchymal status was determined by expression of *PLS3;* Supplementary Figure S3). We can conclude that contamination with white blood cells does occur and might be the source of background *VIM* in *VIM-*negative samples (below maximal expression level observed in healthy controls), but with the applied threshold level for *VIM*-positivity correlation between *CD45* and *VIM* is lost. 

**Figure 5:**
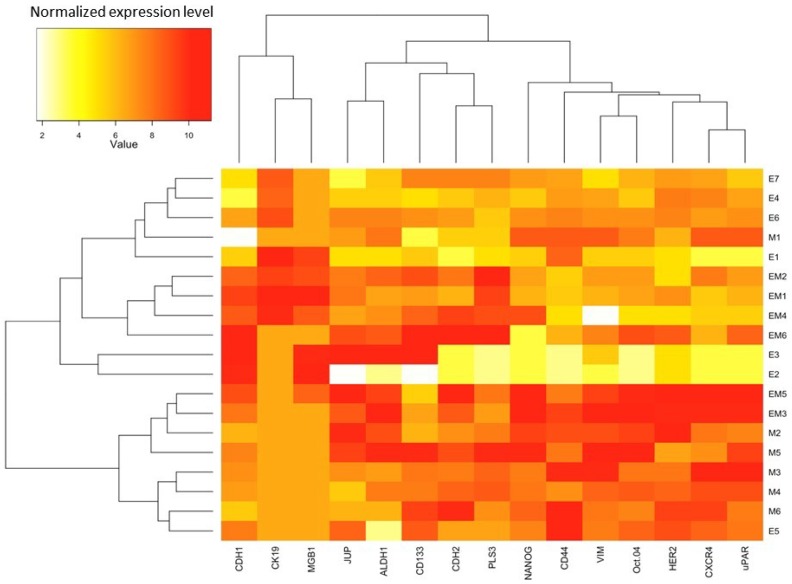
Unsupervised hierarchical clustering of CTCs-positive samples from breast cancer patients with epithelial phenotype (E), mesenchymal phenotype (M) or biphenotypic epithelial-mesenchymal cells (EM). Please note the separation of samples into epithelial-enriched cluster (top samples from E7 to E2) and mesenchymal-enriched cluster (bottom samples from EM5 to E5), with epithelial-mesenchymal samples spread across both groups (and showing more epithelial or more mesenchymal phenotype, depending on the samples).

Hierarchical unsupervised clustering performed in the CTCs-positive samples revealed good separation of epithelial and mesenchymal samples, with biphenotypic group almost equally distributed between epithelial and mesenchymal groups (Figure 5). Top, epithelial cluster was further subdivided based on different expression of *PLS3*, *CDH2*, *CD133*, *ALDH1* and *JUP*, which were normally characteristic for the mesenchymal cluster, what once again underlines the existence of phenotypic CTCs epithelial-mesenchymal plasticity and its functional consequence. Beside classical mesenchymal marker—*VIM*, mesenchymal cluster (lower set of samples) was characterized by high expression of stem cell markers *NANOG*, *CD44*, *OCT-4*, as well as invasion and metastasis related genes *CXCR4* and *uPAR*. It is important to stress that stem cell markers were characteristic for the mesenchymal cluster, except *ALDH1* and *CD133*, which were expressed also in the selected epithelial CTCs samples (Figure 5). 

Such differential grouping of patient samples was further confirmed in principal component analysis. Epithelial and mesenchymal phenotypes were separated, whereas biphenotypic samples were divided between the two clusters (Figure S5).

### 2.2. Correlation with Clinico-Pathological Data

A negative influence of mesenchymal and epithelial-mesenchymal CTCs phenotype was reflected by their association with more aggressive tumour characteristics (Table 1). More than three involved lymph nodes, which corresponds to pathological pN2 stage or higher, were observed in patients with mesenchymal and epithelial-mesenchymal CTCs (*p* = 0.003, Table 1). Patients with T2-4 stage were also overrepresented in the groups with mesenchymal and epithelial-mesenchymal CTCs (*p* = 0.023, 16% and 13%, respectively vs. 5% in the epithelial CTCs group).

**Figure 6:**
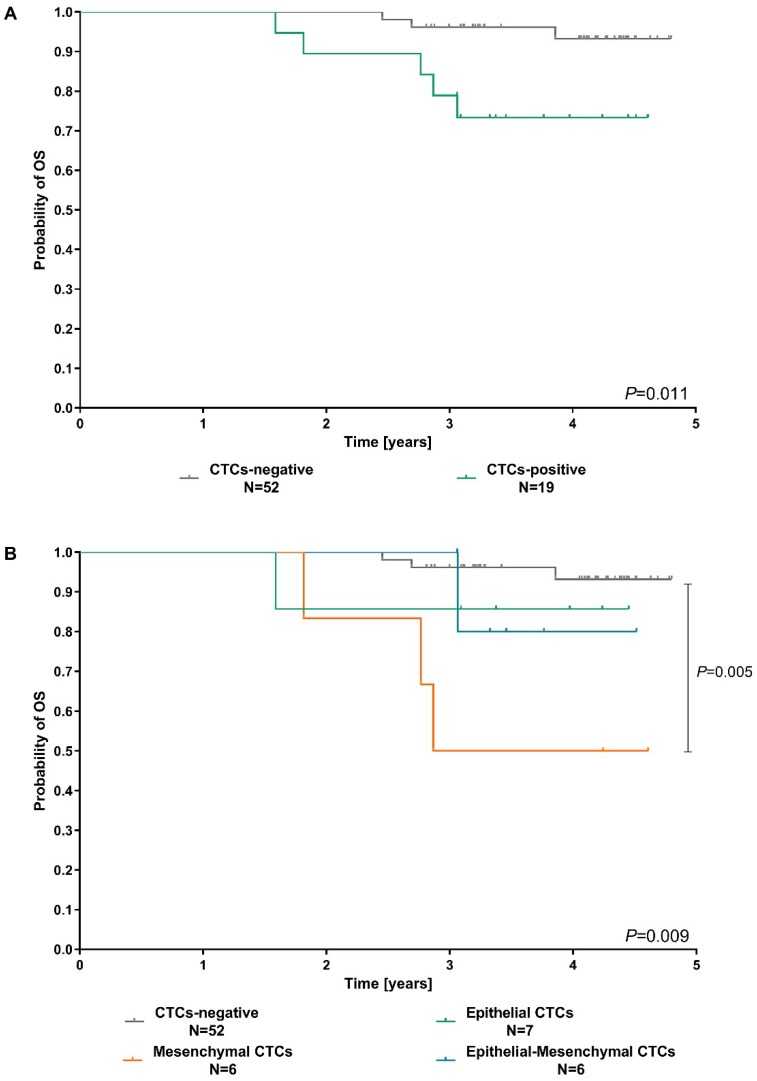
Prognostic significance of CTCs presence (**A**) or CTC phenotype (**B**) in breast cancer patients in terms of overall survival.

Presence of CTCs in peripheral blood was a factor of poor prognosis (Figure 6A). Patients with detected CTCs had significantly shorter overall survival (*p* = 0.011) and higher risk of death, in comparison to patients without CTCs (HR = 5.17, 95% CI 1.23–21.68, *p* = 0.02; Table 2). What is important, phenotype of detected CTCs allowed for more accurate stratification (Figure 6B) than just information about CTCs presence (Figure 6A). Presence of mesenchymal markers in CTCs-EBF correlated with much shorter OS (*p* = 0.005, Figure 6B) in comparison to patients with no CTCs, and higher risk of death (HR = 7.77, 1.85–32.68, *p* = 0.005; Table 2). In the multivariate analysis, including classical histopathological factors assessed in breast cancer, mesenchymal CTCs phenotype (in comparison to all other samples) was an independent predictor of poor outcome (HR = 7.33, 95% CI 1.06–50.41, *p* = 0.04, Table 2). The results of univariate and multivariate analysis of classical histopathological factors are presented in Table S4. As the follow up period in the analysis might be too short to confidently assess the link between CTCs phenotype and deaths, we have included also calculations of relative risk of lymph node involvement as a proximity measure of aggressiveness/metastatic abilities, as it is connected to higher recurrence and mortality rates in breast cancer [54,55,56,57]. Presence of mesenchymal or epithelial-mesenchymal CTCs resulted in 2.17 higher relative risk of lymph node metastases (95% CI 1.32–3.56, *p* = 0.0023, for both) in comparison to samples with no CTCs detected (Table 3). 

Due to the fact that statistics with clinico-pathological data did not require the information regarding expression of epithelial/mesenchymal genes in the CTCs-negative group (as the sample was considered free of CTCs and CTCs phenotyping was not applicable), we could include all CTCs-negative samples (N = 52) in the calculations.

## 3. Discussion

CellSearch is the only FDA-approved method of CTCs detection, with the established prognostic value in a number of different epithelial cancers [58,59,60,61]. The fact that it is based primarily on EpCAM expression on the surface of potential CTCs, has recently raised doubts regarding its sensitivity [8]. EpCAM expression is decreased upon EMT activation [14] and in some aggressive breast cancer cell lines, including normal-like cell lines (e.g., MDA-MB-231, SK-BR-7, BT549), which suggests that EpCAM-based CTCs detection may be insufficient [32,33]. The discovery of molecular heterogeneity of CTCs and its clinical implications highlight the need of improvement of the CTCs isolation methods, which will allow for the maximization of CTCs detection and thereby their further characterization. 

CTCs detection based on expression of a broad range of molecular markers in CTCs-EBF was shown to be a good method of obtaining clinically-informative answers regarding tumour spread [31,62]. Moreover, it is relatively inexpensive, fast, and has a possibility of automation for higher throughput analysis. In the current work, we have performed gene expression profiling of CTCs-EBF obtained by density gradient centrifugation and negative selection with anti-CD45-covered magnetic beads. With this approach it is possible to enrich all phenotypes of CTCs, including epithelial and mesenchymal CTCs [12,23], the latter representing post-EMT state. In the obtained material we have tested a number of markers for the detection of epithelial and mesenchymal CTCs by qPCR. We concluded that contamination of CTCs-EBF with white blood cells (which might express mesenchymal markers) is not influencing the classification of samples as mesenchymal, as long as we applied appropriately set threshold level (above background observed in healthy controls) of mesenchymal markers (*VIM* in particular). In our analysis we noted that about a one third of CTCs-positive samples expressed both epithelial and mesenchymal markers. Simultaneous detection of these markers could be a result of either coexistence of both epithelial and mesenchymal CTCs in the sample or presence of CTCs in the biphenotypic EMT in-transit state, which in the current experimental setting we are unable to distinguish. What is important, both EMT plastic (biphenotypic) CTCs states [25,63] as well as concurrent presence of both epithelial and mesenchymal CTCs in the circulation [64] was shown to be linked to an increased risk of metastases development. In case of biphenotypic CTCs, poor prognosis is explained by the possession of CTCs features which enhance metastases formation, such as a cancer stem cell phenotype, clusters formation or partial epithelial phenotype allowing for faster colonization [63,65]. Presence of both epithelial and mesenchymal CTCs in circulation supports cooperative colonization, during which mesenchymal cells invade and intravasate efficiently, whereas epithelial cells are better at extravasation and colonization [64]. In this context, analysis of CTCs at a single cell level offers great opportunities, as it would allow for precise determination of epithelial/mesenchymal state of each individual cell, providing better insight into CTCs heterogeneity.

Though data point to worse clinical outcome of patients with CTCs expressing mesenchymal markers in metastatic disease [8,17,25,66], in the early disease, mesenchymal phenotype of CTCs remains to be studied in more details. We have observed that 63% of CTCs-positive patients have markers of mesenchymal CTCs, out of which 32% showed mesenchymal markers exclusively (no accompanying epithelial markers expression). With a different set of markers (*VIM* and *TWIST*) Zhang et al. have recently shown that mesenchymal CTCs are detected in 15% of non-metastatic CTCs-positive breast cancer patients, what was significantly lower than in metastatic patients [66]. Nevertheless, mesenchymal CTCs were more accurately predicting the development of distant metastases than any other phenotype (epithelial or epithelial-mesenchymal), and the detection of a single mesenchymal CTCs per 5 mL of blood was related to disease progression (in comparison to 3 CTCs with epithelial-mesenchymal phenotype). With our approach, using a larger panel of markers—*MGB1*, *HER2*, *VIM*, *CDH2*, *PLS3* we have shown that mesenchymal CTCs are prognostic and related to over seven times higher risk of death and two times higher relative risk of developing lymphatic metastases. Though in our study epithelial-mesenchymal phenotype alone was not prognostic in univariate or multivariate analysis, similarly to mesenchymal phenotype it was more frequently found in patients with larger tumours and with involved lymph nodes. It was also associated with worse prognosis than patients with epithelial CTCs. Similar pattern of CTCs phenotype on survival was recently shown by Ou et al., where mesenchymal CTCs predicted the shortest relapse-free survival of patients with hepatocellular carcinoma, followed by biphenotypic and epithelial CTCs [67]. Due to limited number of CTCs positive samples in our study and relatively short overall survival time, further research with larger sample size are required to confirm the clinical significance of CTCs phenotypes according to the established positivity criteria. 

Interestingly, in our analysis we have found two markers—*CDH1* and *CDH2* elevated in samples negative for CTCs markers *MGB1* and *HER2*. Since we have worked with CTCs-enriched blood fractions (contaminated with blood cells being the source of potential background markers expression) we decided to use this more conservative, multimarker-based, classification of samples as CTCs-positive in order to avoid false positive results. It is conceivable that a subpopulation of *CDH1* or *CDH2* positive CTCs lacking *MGB1* and *HER2* could be present in circulation, but it requires additional investigation to identify such CTCs markers. The same problem was shown by Bulfoni et al., who highlighted the lack of appropriate markers for the detection of all CTCs subpopulations [25], and found that apart from epithelial and epithelial-mesenchymal CTCs, subpopulation of CD45-negative cells in blood of breast cancer patients has clinical relevance. What is more, our data indicate that *MGB1* might be downregulated in mesenchymal CTCs samples, thus research relying only on *MGB1* expression for CTCs detection might underestimate the proportion of mesenchymal CTCs in a sample.

We have observed that biphenotypic CTCs-positive samples had increased expression of *PLS3*, which indicates that similarly to colorectal cancer [37], *PLS3* is overexpressed in CTCs undergoing EMT and is a good marker with minimal expression in blood of healthy controls. Moreover, *PLS3* expression was linked with the opposite cadherins expression in the epithelial and mesenchymal CTCs groups. In the epithelial group *PLS3* positively correlated with *CDH2*, characteristic for the EMT state, whereas in the mesenchymal group it was associated with the epithelial *CDH1*. This observation, and the fact that *PLS3* levels were the highest in CTCs samples with epithelial-mesenchymal phenotype, could argued that *PLS3* is the marker of the transitory EMT state, increasing in the cells moving from epithelial to epithelial-mesenchymal state and decreasing in cells moving from epithelial-mesenchymal to mesenchymal state. In that case, increasing *PLS3* would correlate with increasing *CDH2* in the epithelial CTCs committed to EMT, and in the mesenchymal cells finishing EMT, *PLS3* would correlate with the decreasing *CDH1* levels. 

## 4. Materials and Methods

Peripheral blood samples (5 mL) for CTCs enrichment were collected from 83 previously untreated patients with operable, non-lobular breast cancer (stage I-III) admitted to the Medical University of Gdansk between April 2011 and May 2013. Informed consent was collected from all participants included in the study, and permission was granted by the Bioethical Committee of the Medical University of Gdansk.

As controls, blood samples from 22 healthy female volunteers with no prior cancer history were similarly drawn and processed. Median age of patients was 61.9 years (range 39.1–82.6 years) and median age of the healthy controls was 50.2 years (range 21–74.1 years). Median follow-up period was 4.1 years and was last updated in February 2016. Informed consent was collected from all participants included in the study, and permission was granted by the Bioethical Committee of the Medical University of Gdansk (NKEBN/30/2010, approved on the 17th March 2010). Clinico-pathological characteristics of the studied group are presented in Table 4. Tumour grade was assessed according to the modified Bloom and Richardson system. Estrogen and progesterone receptors statuses were evaluated according to the Allred system with cut-point 3 for positive result. HER2 positivity was based on standard criteria: 3+ in immunohistochemistry or positive result in fluorescent in situ hybridization.

### 4.1. Gene Expression Analysis

Peripheral blood samples were collected to EDTA-coated tubes and enriched for CTCs, as described before [12] and presented on the Supplementary Figure S6. Briefly, density gradient centrifugation was performed followed by depletion of cells of hematopoietic origin with anti-CD45-covered immunomagnetic beads (CD45 Dynabeds, Invitrogen, Carlsbad, CA, USA). From such CTCs-EBF RNA was isolated using TRIzol Reagent (Invitrogen). Up to 10 µL of total RNA was used in a reverse transcription reaction (Transcriptor First Strand Synthesis Kit, Roche, Basel, Switzerland) with random hexamers according to manufacturer’s protocol. Quality of the obtained samples was tested by the analysis of expression of two reference genes—*GAPDH* (Hs99999905_m1) and *YWHAZ* (Hs03044281_g1) in qPCR, followed by the analysis of cytokeratin 19 (*CK19*, Hs01051611_gH), vimentin (*VIM*, Hs00185584_m1), mammaglobin-1 (*MGB1*, also known as *SCGB2A2*, Hs00935948_m1), *HER2*, and invasion-related genes (*CXCR4*, Hs00237052_m1; *uPAR*, also known as *PLAUR*, Hs00182181_m1) in qPCR (CFX cycler, Bio-Rad) as previously described [12] using commercially available TaqMan probes and Universal PCR Master Mix (Applied Biosystems, Foster City, CA, USA). Additionally, to control for contamination with white blood cells expression of CD45 was tested (Hs04189704_m1). qPCR were performed in duplicates on 96-well plates in the following conditions: 2 min at 60 °C, 10 min at 95 °C, 45 cycles of 1 min at 60 °C and 15 s at 95 °C. Results were analyzed in a relative manner using modified ΔΔCt approach in qBase software (Biogazelle, Zwijnaarde, Belgium) [68]. Briefly, based on the average Cq, gene expression level was calculated in relation to two reference genes (*GAPDH* and *YWHAZ*), a calibrator (sample with the lowest measurable expression of a given gene) and additionally corrected for run-to-run variation by comparing the expression level of each gene in an inter-run calibrator (mix of cDNA of healthy breast tissue) present on every plate. Thus, sample with the smallest expression level has the expression level of one, if no expression was detected in qPCR, expression level equalled to zero.

Because of low amount of isolated RNA or its insufficient quality, more detailed gene expression analysis was possible in 46 out of 83 collected CTCs-EBF samples. After optimizing targeted cDNA preamplification, according to the protocol described before [23], 1 µL of cDNA was preamplified in TaqMan PreAmp Master Mix 2x (Applied Biosystems) with the following TaqMan assays pooled - E-cadherin (also known as *CDH1*, Hs01023894_m1), N-cadherin (also known as *CDH2*, Hs00983056_m1), plastin-3 (*PLS3*, Hs00958354_m1), *CD44* (Hs01075862_m1), *NANOG* (Hs02387400_g1), *ALDH1* (Hs00946916_m1), *OCT-4* (also known as *POU5F1*, Hs00999632_g1), *CD133* (also known as *PROM1*, Hs01009250_m1), plakoglobin (*JUP*, Hs00158408_m1) and two reference genes: *GAPDH* and *YWHAZ*.

Preamplification was performed in 50 µL (2 × 25 µL) according to the protocol: 10 min at 95 °C, 10 cycles of 15 s at 95 °C and 4 min at 60 °C. After 10 cycles no amplification bias was detected. qPCR cycling parameters were the same as for non-preamplified samples. Gene expression levels were scaled to the samples with lowest expression level. CTCs-EBF were considered positive for a given marker when expression of that marker was higher than the expression detected in the control samples from healthy donors (Supplementary Table S2). Samples were classified as CTCs positive when at least one of mammary epithelial transcripts (*MGB1* or *HER2*) was detected, then further subdivided into EMT classes based on the expression of epithelial (*CK19*, *CDH1*) and mesenchymal (*VIM*, *CDH2*, *PLS3*) markers. Detailed division into classes is shown on Figure 1. 

### 4.2. Statistical Analysis

Categorical variables were compared by χ2 contingency tables and continuous variables were compared by Spearman’s rank order test. Kruskal-Wallis test was used to examine differences between quantitative values (gene expression level). Kaplan-Meier curves for overall survival (OS) were compared using F-Cox test (or log-rank test in case of OS in *CDH2-*positive and –negative samples in CTCs-negative group). OS was defined as the time from surgery to death or censoring (patient lost to follow-up or alive without relapse at the end of follow up period). Hazard ratios (HRs) with 95% confidence intervals (95% CI) were computed using Cox regression analysis. Significance was defined as *p* < 0.05. STATISTICA software version 13.0 (StatSoft, Cracov, Poland) for Windows was used for all statistical analyses. Hierarchical clustering and principal component analysis were performed on normalized data in R software (R Development Core Team). Relative risks were calculated using on line Medcalc tool. Figures have been prepared in GraphPad Prism software version 8 (GraphPad Software, San Diego, CA, USA).

## 5. Conclusions

Our results have shown that different EMT phenotypes of CTCs (epithelial, epithelial-mesenchymal and mesenchymal) can be distinguished in CTCs-EBF of early breast cancer using a panel of 7 genes (*MGB1/HER2/CK19/CDH1/CDH2/VIM/PLS3*) tested in qPCR. For the assessment of mesenchymal genes in CTCs-enriched blood fractions careful selection of a healthy control cut-off level is required to avoid false-positive results. CTCs phenotypes can provide clinically important information regarding patients’ survival, especially mesenchymal phenotype, which is related to significantly decreased overall survival. Epithelial-mesenchymal phenotype, correlating with poor clinico-pathological characteristics, did not result in significantly decreased survival, what might be the result of its heterogeneous advancement in the EMT program. Our research provides evidence showing the translational benefits of molecular profiling of CTCs, rather than performing just CTCs enumeration. Further studies, including larger number of samples and longer follow up period are required to confirm the clinical significance of molecular profiling of CTCs according to the established positivity criteria.

## Figures and Tables

**Table 1:** Correlations between clinico-pathological characteristics and detected phenotype of CTCs in breast cancer patients. Statistically significant (*p* < 0.05) results are shown in bold.

Variable	Status	N	CTCs-Negative	Number of Cases with a Given CTCs Phenotype *			*p*
				E	M	EM	
T stage	T1	32	26 (81%)	5 (3%)	0 (0%)	1 (16%)	**0.023**
	T2-T4	38	25 (66%)	2 (5%)	6 (16%)	5 (13%)	
N stage	N−	37	32 (86%)	3 (8%)	1 (3%)	1 (3%)	**0.043**
	N+	34	20 (59%)	4 (11%)	5 (15%)	5 (15%)	
Lymph nodes involved	1–3 (pN1)	59	46 (78%)	7 (12%)	2 (3%)	4 (7%)	**0.003**
	≥4 (≥pN2)	12	6 (50%)	0 (0%)	4 (33%)	2 (17%)	
Grading	G1-G2	45	33 (73%)	7 (16%)	3 (7%)	2 (4%)	0.077
	G3	26	19 (73%)	0 (0%)	3 (12%)	4 (15%)	
ER status	negative	15	12 (80%)	1 (7%)	2 (13%)	0 (0%)	0.483
	positive	56	40 (71%)	6 (11%)	4 (7%)	6 (11%)	
PR status	negative	16	13 (82%)	1 (6%)	1 (6%)	1 (6%)	0.875
	positive	55	39 (71%)	6 (11%)	5 (9%)	5 (9%)	
HER2 status	negative	50	37 (74%)	7 (14%)	4 (8%)	2 (4%)	0.060
	positive	19	13 (67%)	0 (0%)	2 (11%)	4 (22%)	

* E—epithelial phenotype, M—mesenchymal phenotype, EM—epithelial-mesenchymal phenotype. Percentages were counted relative to all samples.

**Table 2:** Univariate and multivariate analysis showing risk of death of breast cancer patients (N = 72) depending on the CTCs phenotype (vs. all other samples). Statistically significant (*p* < 0.05) results are shown in bold.

Variable–CTCs Phenotype	Univariate Analysis			Multivariate Analysis		
	HR	95% CI	*p*	HR	95% CI	*p*
Epithelial	1.40	0.17–11.36	0.75	1.13	0.12–10.61	0.92
Mesenchymal	7.77	1.85–32.68	**0.005**	7.33	1.06–50.41	**0.04**
Epithelial-mesenchymal	1.65	0.20–13.59	0.64	0.95	0.10–8.75	0.97
Any CTCs	5.17	1.23–21.68	**0.02**	3.40	0.69–16.69	0.13

**Table 3:** Relative risk of lymph node involvement depending on the phenotype of CTCs detected (vs. no CTCs). Statistically significant (*p* < 0.05) results are shown in bold.

Variable–CTCs Phenotype	Relative Risk	95% CI	*p*
Epithelial	1.49	0.72–3.08	0.29
Mesenchymal	2.17	1.32–3.56	**0.0023**
Epithelial-mesenchymal	2.17	1.32–3.56	**0.0023**
Any CTCs	1.92	1.24–2.96	**0.0035**

**Table 4:** Clinico-pathological characteristics of the studied group of breast cancer patients (N = 83).

Variable	Status	Number of Cases	%
Age	<50 year	18	21.7
	≥50 year	65	78.3
T stage	T1	39	47.0
	T2	38	45.8
	T3	3	3.6
	T4	2	2.4
	Missing data	1	1.2
N stage	N+	42	50.6
	N−	41	49.4
Grading	G1	12	14.5
	G2	43	51.8
	G3	28	33.7
ER status	Positive	66	79.5
	Negative	17	20.5
PR status	Positive	65	78.3
	Negative	18	21.7
HER2 status	Positive	20	24.1
	Negative	61	73.5
	Missing data	2	2.4

## Supplementary Materials

The following are available online at http://www.mdpi.com/2072-6694/11/1/59/s1, Figure S1: Relative expression level of genes used for CTCs-positivity determination and distribution into classes, invasion-related genes, stem cell and cluster formation markers in CTCs-negative, CTCs-positive samples and in healthy controls. Statistically significant differences are marked (* *p* < 0.05, ** *p* < 0.01, *** *p* < 0.001), Figure S2: Prognostic significance (overall survival) of *CDH1* (A) and *CDH2* (B) in CTCs-EBF classified as CTCs-negative due to lack of expression of basic CTCs markers (*MGB1* and *HER2*), Figure S3: Marker status in each CTCs sample from epithelial (E), mesenchymal (M) and epithelial-mesenchymal (EM) group. Positive marker status is shown in orange, negative in blue, Figure S4: Estimation of leukocyte contamination in CTCs-EBF. Relative expression level of *CD45* in breast cancer patients (N = 39) and healthy controls (N = 9), Figure S5: Principal component analysis showing separate grouping of CTCs-positive samples classified as epithelial (E) or mesenchymal (M); epithelial-mesenchymal (EM) samples are divided between epithelial and mesenchymal samples, Figure S6. Blood samples processing and gene expression analysis scheme. Table S1. List of genes tested describing their function. Table S2: Relative expression level of all genes tested in qPCR in CTCs-positive breast cancer patients (N = 19) and healthy controls (N = 22), Table S3: Spearman correlations coefficients (ρ_S_) between *CD45* expression and mesenchymal markers (*VIM*, *CDH2* and *PLS3*) analyzed in CTCs-enriched blood fractions of breast cancer patients. CTCs positive samples correlations are subdivided into (i) marker-positive and marker-negative samples based on the maximal cut-off level recorded in healthy controls; or (ii) into phenotypes of CTCs (epithelial, mesenchymal, epithelial-mesenchymal). Statistically significant (*p* < 0.05) results are marked in bold, Table S4: Univariate and multivariate analysis showing the risk of death of breast cancer patients depending on the clinical variables.
