# Supplementary material for: Spectrum of Epithelial-Mesenchymal Transition Phenotypes in Circulating Tumour Cells from Early Breast Cancer Patients
Source: Cancers (Basel). 2019 Jan 9;11(1):59. doi: 10.3390/cancers11010059 (PMC6356662; doi:10.3390/cancers11010059)
Supplement: Supplementary file 1 [file cancers-11-00059-s001.pdf]

# Supplementary Materials: Spectrum of Epithelial-Mesenchymal Transition Phenotypes in Circulating Tumour Cells from Early Breast Cancer Patients

**Aleksandra Markiewicz, Justyna Topa, Anna Nagel, Jaroslaw Skokowski, Barbara Seroczynska, Tomasz Stokowy, Marzena Welnicka-Jaskiewicz and Anna J. Zaczek**

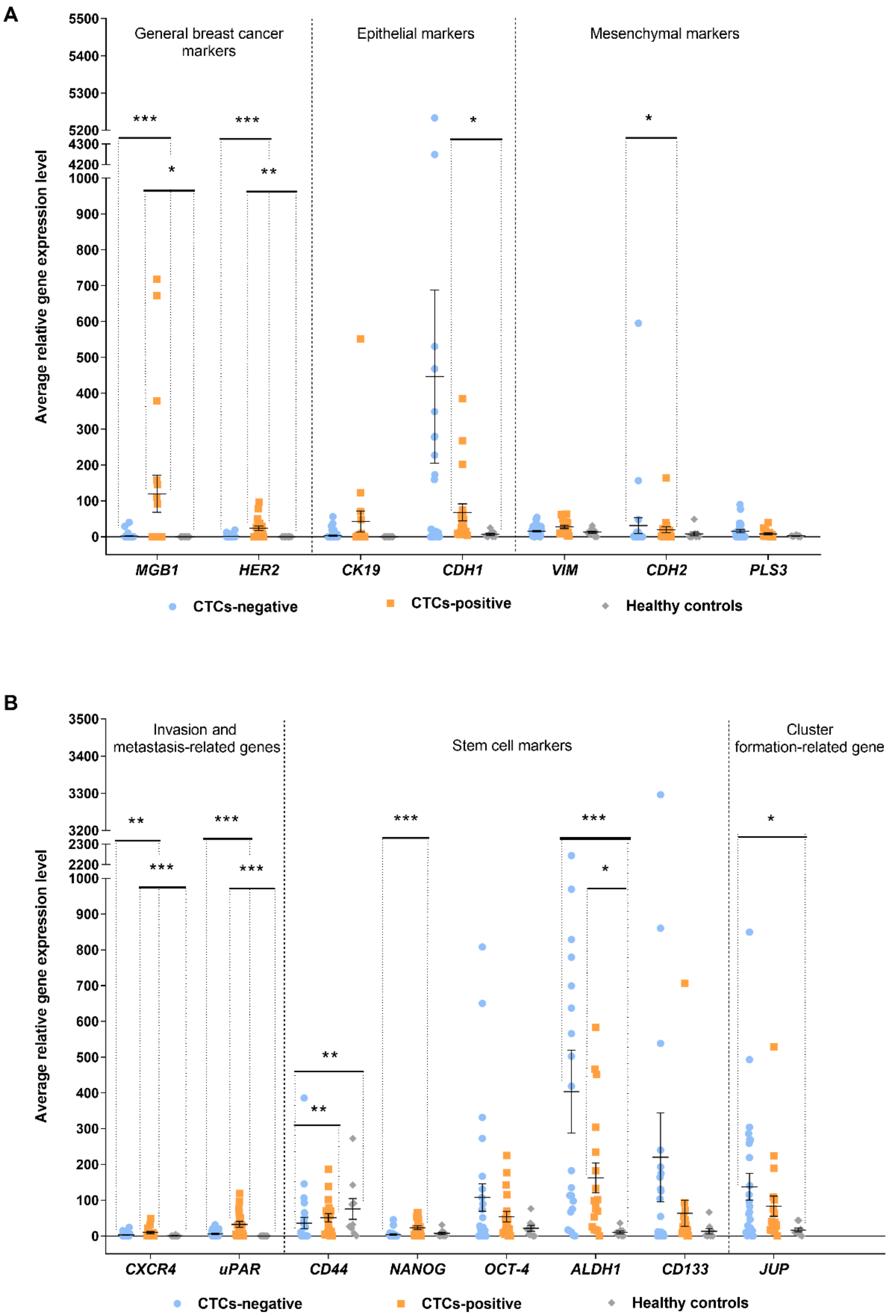


**Figure S1.** Relative expression level of genes used for CTCs-positivity determination and distribution into EMT classes (**A**), invasion-related genes, stem cell and cluster formation markers (**B**) in CTCs-negative, CTCs-positive samples and in healthy controls. Statistically significant differences are marked (* $p < 0.05$, ** $p < 0.01$, *** $p < 0.001$).

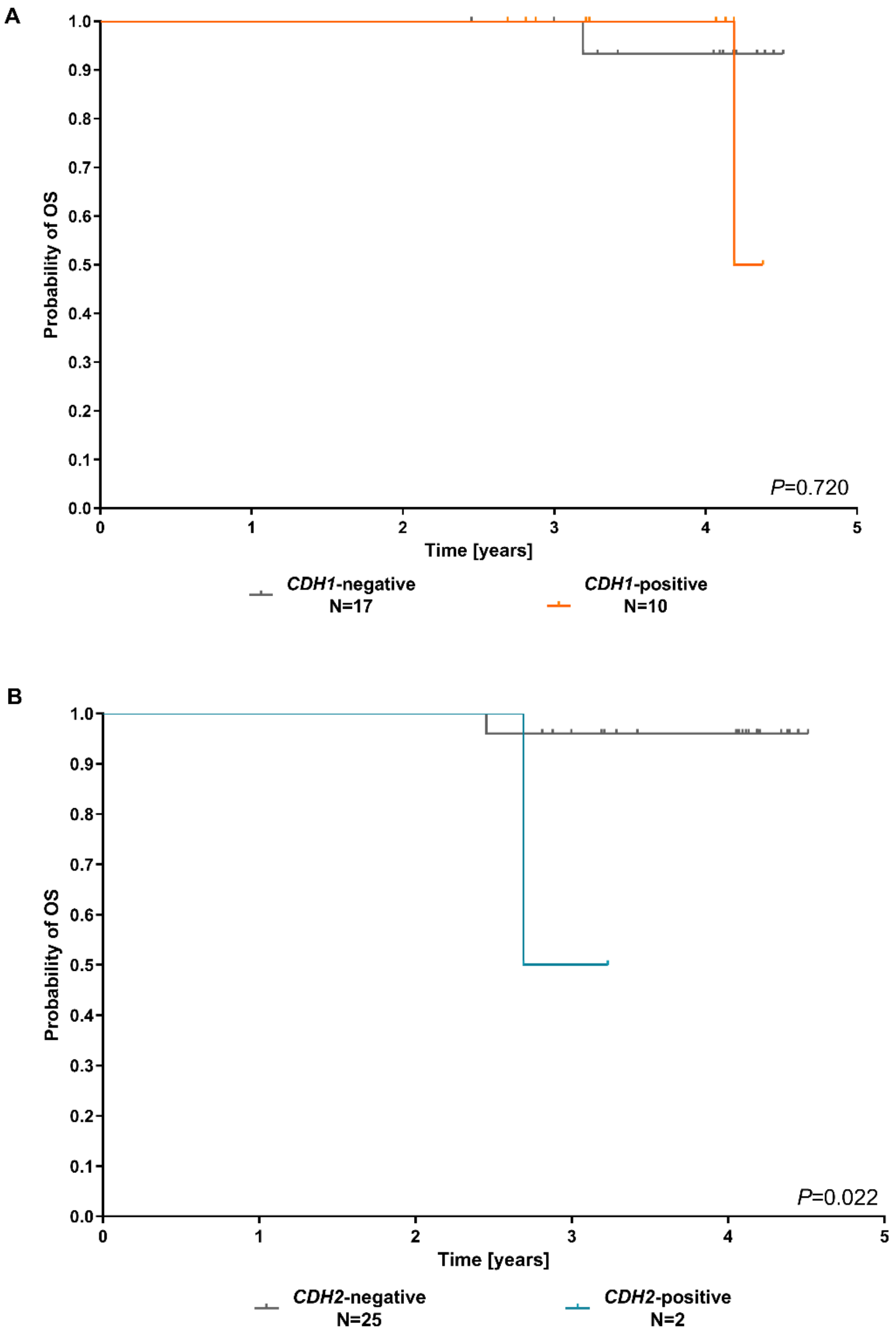


**Figure S2.** Prognostic significance (overall survival) of *CDH1* (**A**) and *CDH2* (**B**) in CTCs-EBF classified as CTCs-negative due to lack of expression of basic CTCs markers (*MGB1* and *HER2*).

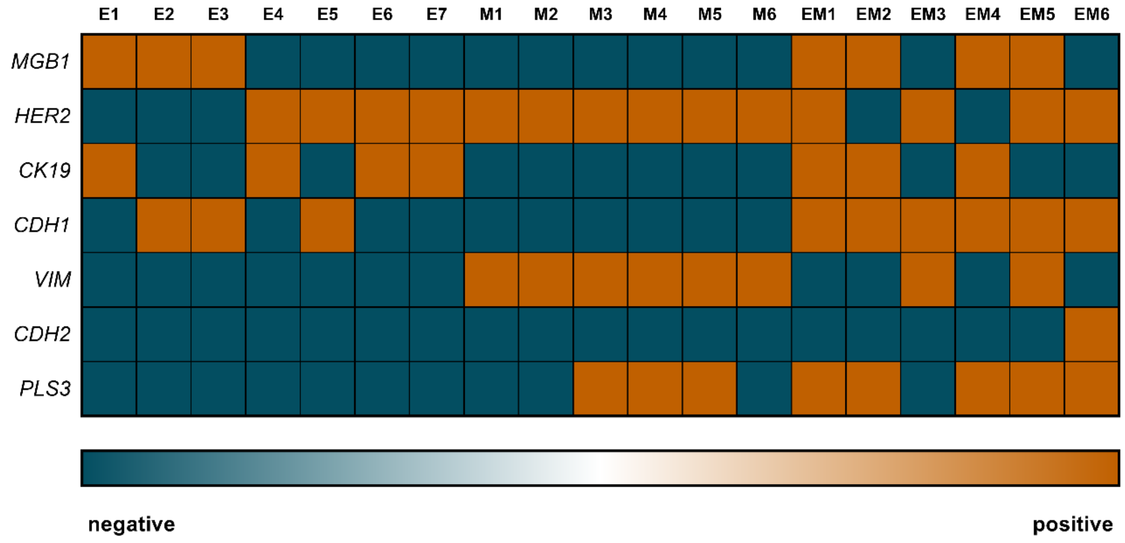


**Figure S3.** Marker status in each CTCs sample from epithelial (E), mesenchymal (M) and epithelial-mesenchymal (EM) group. Positive marker status is shown in orange, negative in blue.

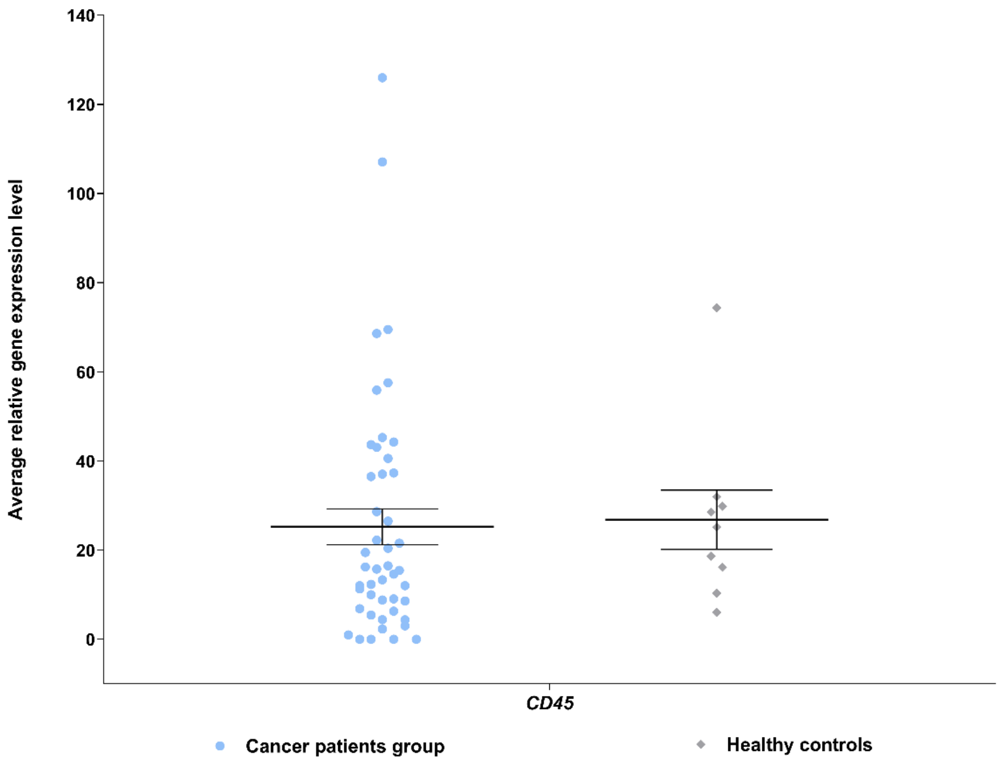


**Figure S4.** Estimation of leukocyte contamination in CTCs-EBF. Relative expression level of *CD45* in breast cancer patients (N = 39) and healthy controls (N = 9).

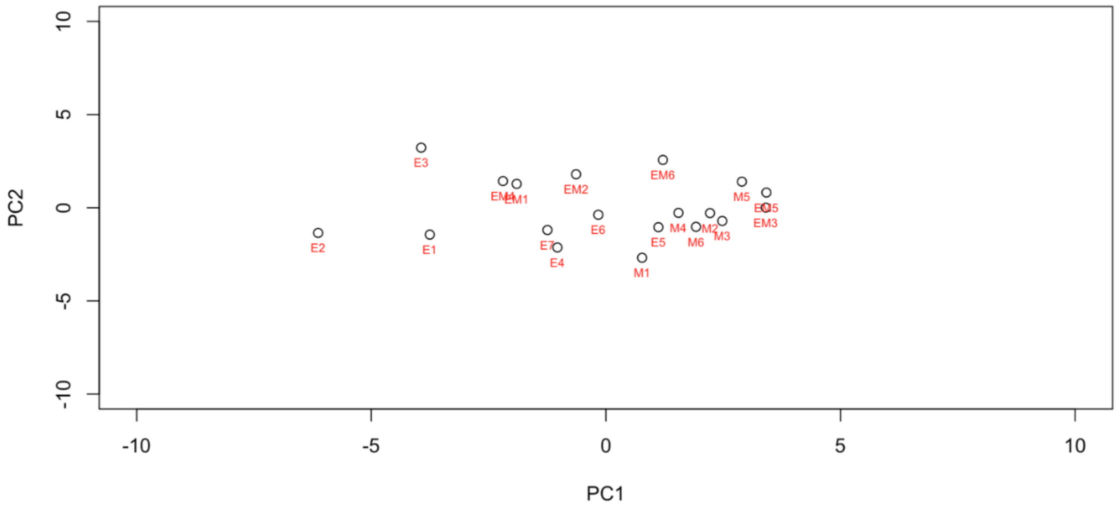


**Figure S5.** Principal component analysis showing separate grouping of CTCs-positive samples classified as epithelial (E) or mesenchymal (M); epithelial-mesenchymal (EM) samples are divided between epithelial and mesenchymal samples.

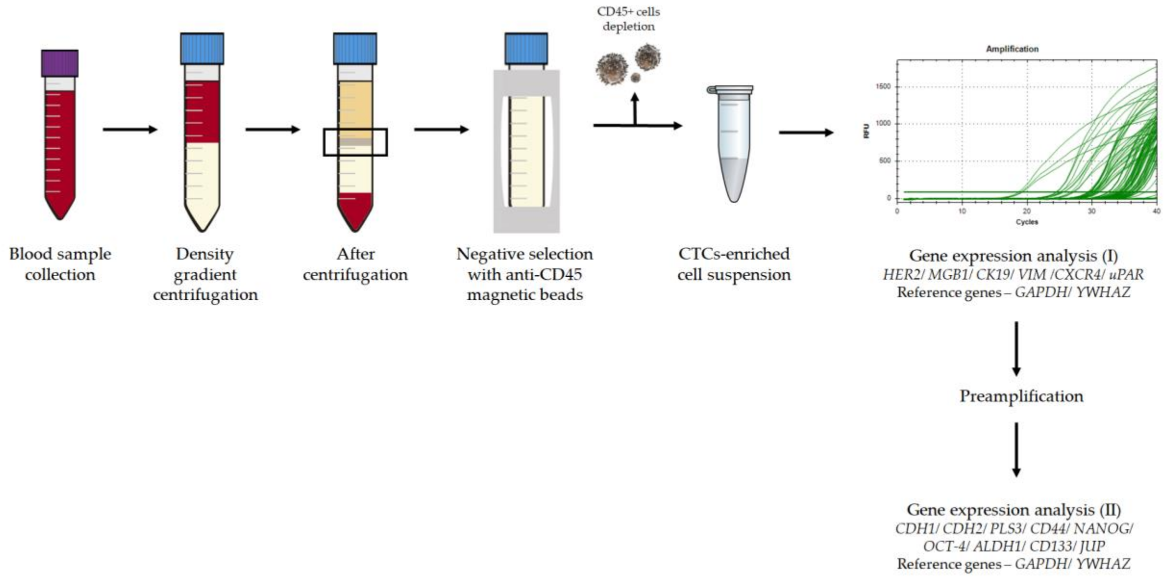


**Figure S6.** Blood samples processing and gene expression analysis scheme.

**Table S1.** List of genes describing their functions.

| Type of Marker | Marker (full name) | Abbreviation | Gene | Function in Cancer and Prognostic Value | References |
|---|---|---|---|---|---|
| General Breast Cancer markers | Mammaglobin-1 (Mammaglobin-A/ Secretoglobin family 2A member 2) | MGB1 | *SCGB2A2* | Secretory protein exclusively expressed in cells originating from mammary gland and in some breast cancer cell lines. | [1] |
| | Receptor tyrosine-protein kinase erbB-2 | HER2 | *ERBB2* | Tyrosine-protein kinase growth factor receptor overexpressed in 20-30% of primary breast cancers. Proto-oncogene in breast cancer. | [2,3] |
| Epithelial markers | Cytokeratin-19 (Keratin, type 1 cytoskeletal 19) | CK19 | *KRT19* | Intracellular intermediate filament constituting cytoskeleton of epithelial cells. | [4] |
| | E-cadherin | ECAD | *CDH1* | Transmembrane glycoprotein mediating intercellular adhesion. Its loss in tumors as a result of EMT contributes to metastatic dissemination. Re-expressed in metastatic sites. Involved in tumour cells clusters formation. | [5–7] |
| Mesenchymal markers | Vimentin | VIM | *VIM* | Intracellular intermediate filament of mesenchymal cells, constituting their cytoskeleton. | [8] |
| | N-cadherin | NCAD | *CDH2* | Transmembrane protein expressed in cells of mesenchymal origin, mediates cell-cell adhesion. Its increased expression is correlated with downregulation of E-cadherin in so called cadherin switch. Forms weaker adherent junctions between adjacent cells, what increases their motility. | [9,10] |

| | | | | | |
|---|---|---|---|---|---|
| | Plastin-3 | PLS3 | *PLS3* | Potential marker of breast and colorectal cancer CTCs in all EMT stages. Enables tumour to form metastases, avoid anoikis and survive during therapy. Its high expression in human cancer cell lines correlate with mesenchymal, stemness and metastasis-related genes expression. | [11,12] |
| Invasion and metastasis-related genes | C-X-C chemokine receptor type 4 | CXCR4 | *CXCR4* | Protein overexpressed in breast cancer cell lines, involved in determination of metastatic destination of tumour cells and their migration. | [13] |
| | Urokinase (uPA) receptor | uPAR | *PLAUR* | Part of uPA system, which is involved in processes causing metastases. Overexpressed in malignant tumours in contrast to corresponding normal tissues. Its high level in tumours (among others in BCs) correlates with poor prognosis. | [14,15] |
| Stem cell markers | CD44 molecule | CD44 | *CD44* | Hyaluronan receptor involved in interaction with tumour stroma, extravasation, migration, survival and apoptosis resistance. Upregulated in cells with stem cells properties. | [16–18] |
| | Homeobox protein NANOG | NANOG | *NANOG* | Transcription factor positively regulating EMT and promoting tumorigenesis and metastasis. Required for maintaining the pluripotency of embryonic stem cells. | [19–21] |
| | Aldehyde dehydrogenase 1 family member A | ALDH1 | *ALDH1A1* | Upregulated in mammary epithelial cells with stem properties. Associated with increased tumorigenesis. | [22,23] |
| | CD133 molecule (prominin-1) | CD133 | *PROM-1* | Transmembrane protein associated with maintaining stem cell properties. Factor of poor prognosis in BC. | [24,25] |

| | | | | | |
|---|---|---|---|---|---|
| | Octamer-binding transcription factor 4 (POU domain, class 5, transcription factor 1) | OCT-4 | *POU5F1* | Involved in maintenance of self-renewal in embryonic stem cells. Expressed in human BC stem-like cells. Induces expression of NANOG and promotes EMT. | [19,21,26,27] |
| Cluster formation marker | Plakoglobin (junction plakoglobin/ gamma-catenin) | PKBG | *JUP* | Directly involved in tumour spread and CTCs-clusters formation. Its high expression in PT in BC patients is a factor of poor prognosis. | [7,28] |

1. Watson, M.A.; Fleming, T.P. Mammaglobin, a mammary-specific member of the uteroglobin gene family, is overexpressed in human breast cancer. *Cancer Res.* **1996**, *56*, 860–865.
2. Toikkanen, S.; Helin, H.; Isola, J.; Joensuu, H. Prognostic significance of HER-2 oncoprotein expression in breast cancer: a 30-year follow-up. *J. Clin. Oncol.* **1992**, *10*, 1044–1048, doi:10.1200/JCO.1992.10.7.1044.
3. Slamon, D.J.; Clark, G.M.; Wong, S.G.; Levin, W.J.; Ullrich, A.; McGuire, W.L. Human breast cancer: correlation of relapse and survival with amplification of the HER-2/neu oncogene. *Science* **1987**, *235*, 177–182.
4. Coulombe, P.A.; Omary, M.B. 'Hard' and 'soft' principles defining the structure, function and regulation of keratin intermediate filaments. *Curr. Opin. Cell Biol.* **2002**, *14*, 110–122.
5. Onder, T.T.; Gupta, P.B.; Mani, S.A.; Yang, J.; Lander, E.S.; Weinberg, R.A. Loss of E-cadherin promotes metastasis via multiple downstream transcriptional pathways. *Cancer Res.* **2008**, *68*, 3645–3654, doi:10.1158/0008-5472.CAN-07-2938.
6. Bukholm, I.K.; Nesland, J.M.; Børresen-Dale, A.L. Re-expression of E-cadherin, alpha-catenin and beta-catenin, but not of gamma-catenin, in metastatic tissue from breast cancer patients [seecomments]. *J. Pathol.* **2000**, *190*, 15–19, doi:10.1002/(SICI)1096-9896(200001)190:1<15::AID-PATH489>3.0.CO;2-L.
7. Aceto, N.; Bardia, A.; Miyamoto, D.T.; Donaldson, M.C.; Wittner, B.S.; Spencer, J.A.; Yu, M.; Pely, A.; Engstrom, A.; Zhu, H., et al. Circulating tumor cell clusters are oligoclonal precursors of breast cancer metastasis. *Cell* **2014**, *158*, 1110–1122, doi:10.1016/j.cell.2014.07.013.
8. Herrmann, H.; Aebi, U. Intermediate filaments and their associates: multi-talented structural elements specifying cytoarchitecture and cytodynamics. *Curr. Opin. Cell Biol.* **2000**, *12*, 79–90.
9. Aiello, N.M.; Maddipati, R.; Norgard, R.J.; Balli, D.; Li, J.; Yuan, S.; Yamazoe, T.; Black, T.; Sahmoud, A.; Furth, E.E., et al. EMT Subtype Influences Epithelial Plasticity and Mode of Cell Migration. *Dev. Cell* **2018**, *45*, 681–695.e684, doi:10.1016/j.devcel.2018.05.027.
10. Thiery, J.P.; Acloque, H.; Huang, R.Y.; Nieto, M.A. Epithelial-mesenchymal transitions in development and disease. *Cell* **2009**, *139*, 871–890, doi:10.1016/j.cell.2009.11.007.
11. Ueo, H.; Sugimachi, K.; Gorges, T.M.; Bartkowiak, K.; Yokobori, T.; Müller, V.; Shinden, Y.; Ueda, M.; Mori, M.; Kuwano, H., et al. Circulating tumour cell-derived plastin3 is a novel marker for predicting long-term prognosis in patients with breast cancer. *Br. J. Cancer* **2015**, *112*, 1519–1526, doi:10.1038/bjc.2015.132.
12. Yokobori, T.; Iinuma, H.; Shimamura, T.; Imoto, S.; Sugimachi, K.; Ishii, H.; Iwatsuki, M.; Ota, D.; Ohkuma, M.; Iwaya, T., et al. Plastin3 is a novel marker for circulating tumor cells undergoing the epithelial-mesenchymal transition and is associated with colorectal cancer prognosis. *Cancer Res.* **2013**, *73*, 2059–2069, doi:10.1158/0008-5472.CAN-12-0326.
13. Müller, A.; Homey, B.; Soto, H.; Ge, N.; Catron, D.; Buchanan, M.E.; McClanahan, T.; Murphy, E.; Yuan, W.; Wagner, S.N., et al. Involvement of chemokine receptors in breast cancer metastasis. *Nature* **2001**, *410*, 50–56, doi:10.1038/35065016.
14. Shimizu, M.; Cohen, B.; Goldvasser, P.; Berman, H.; Virtanen, C.; Reedijk, M. Plasminogen activator uPA is a direct transcriptional target of the JAG1-Notch receptor signaling pathway in breast cancer. *Cancer Res.* **2011**, *71*, 277–286, doi:10.1158/0008-5472.CAN-10-2523.
15. Stillfried, G.E.; Saunders, D.N.; Ranson, M. Plasminogen binding and activation at the breast cancer cell surface: the integral role of urokinase activity. *Breast Cancer Res.* **2007**, *9*, R14, doi:10.1186/bcr1647.
16. Zöller, M. CD44: can a cancer-initiating cell profit from an abundantly expressed molecule? *Nat Rev Cancer* **2011**, *11*, 254–267, doi:10.1038/nrc3023.

17. Mani, S.A.; Guo, W.; Liao, M.J.; Eaton, E.N.; Ayyanan, A.; Zhou, A.Y.; Brooks, M.; Reinhard, F.; Zhang, C.C.; Shipitsin, M., et al. The epithelial-mesenchymal transition generates cells with properties of stem cells. *Cell* **2008**, *133*, 704–715, doi:10.1016/j.cell.2008.03.027.
18. McFarlane, S.; Coulter, J.A.; Tibbits, P.; O'Grady, A.; McFarlane, C.; Montgomery, N.; Hill, A.; McCarthy, H.O.; Young, L.S.; Kay, E.W., et al. CD44 increases the efficiency of distant metastasis of breast cancer. *Oncotarget* **2015**, *6*, 11465–11476, doi:10.18632/oncotarget.3410.
19. Wang, D.; Lu, P.; Zhang, H.; Luo, M.; Zhang, X.; Wei, X.; Gao, J.; Zhao, Z.; Liu, C. Oct-4 and Nanog promote the epithelial-mesenchymal transition of breast cancer stem cells and are associated with poor prognosis in breast cancer patients. *Oncotarget* **2014**, *5*, 10803–10815, doi:10.18632/oncotarget.2506.
20. Lu, X.; Mazur, S.J.; Lin, T.; Appella, E.; Xu, Y. The pluripotency factor nanog promotes breast cancer tumorigenesis and metastasis. *Oncogene* **2014**, *33*, 2655–2664, doi:10.1038/onc.2013.209.
21. Chambers, I.; Colby, D.; Robertson, M.; Nichols, J.; Lee, S.; Tweedie, S.; Smith, A. Functional expression cloning of Nanog, a pluripotency sustaining factor in embryonic stem cells. *Cell* **2003**, *113*, 643–655.
22. Deng, Y.; Zhang, Y.; Sun, S.; Wang, Z.; Wang, M.; Yu, B.; Czajkowsky, D.M.; Liu, B.; Li, Y.; Wei, W., et al. An integrated microfluidic chip system for single-cell secretion profiling of rare circulating tumor cells. *Sci. Rep.* **2014**, *4*, 7499, doi:10.1038/srep07499.
23. Ginestier, C.; Hur, M.H.; Charafe-Jauffret, E.; Monville, F.; Dutcher, J.; Brown, M.; Jacquemier, J.; Viens, P.; Kleer, C.G.; Liu, S., et al. ALDH1 is a marker of normal and malignant human mammary stem cells and a predictor of poor clinical outcome. *Cell Stem Cell* **2007**, *1*, 555–567, doi:10.1016/j.stem.2007.08.014.
24. Liu, T.J.; Sun, B.C.; Zhao, X.L.; Zhao, X.M.; Sun, T.; Gu, Q.; Yao, Z.; Dong, X.Y.; Zhao, N.; Liu, N. CD133+ cells with cancer stem cell characteristics associates with vasculogenic mimicry in triple-negative breast cancer. *Oncogene* **2013**, *32*, 544–553, doi:10.1038/onc.2012.85.
25. Li, Z.; Yin, S.; Zhang, L.; Liu, W.; Chen, B.; Xing, H. Clinicopathological characteristics and prognostic value of cancer stem cell marker CD133 in breast cancer: a meta-analysis. *Onco. Targets Ther.* **2017**, *10*, 859–870, doi:10.2147/OTT.S124733.
26. Ponti, D.; Costa, A.; Zaffaroni, N.; Pratesi, G.; Petrangolini, G.; Coradini, D.; Pilotti, S.; Pierotti, M.A.; Daidone, M.G. Isolation and in vitro propagation of tumorigenic breast cancer cells with stem/progenitor cell properties. *Cancer Res.* **2005**, *65*, 5506–5511, doi:10.1158/0008-5472.CAN-05-0626.
27. Chiou, S.H.; Wang, M.L.; Chou, Y.T.; Chen, C.J.; Hong, C.F.; Hsieh, W.J.; Chang, H.T.; Chen, Y.S.; Lin, T.W.; Hsu, H.S., et al. Coexpression of Oct4 and Nanog enhances malignancy in lung adenocarcinoma by inducing cancer stem cell-like properties and epithelial-mesenchymal transdifferentiation. *Cancer Res.* **2010**, *70*, 10433–10444, doi:10.1158/0008-5472.CAN-10-2638.
28. Goto, W.; Kashiwagi, S.; Asano, Y.; Takada, K.; Takahashi, K.; Hatano, T.; Takashima, T.; Tomita, S.; Motomura, H.; Ohsawa, M., et al. Circulating tumor cell clusters-associated gene plakoglobin is a significant prognostic predictor in patients with breast cancer. *Biomark Res.* **2017**, *5*, 19, doi:10.1186/s40364-017-0099-2.

**Table S2.** Relative expression level of all genes tested in qPCR in CTCs-positive breast cancer patients (N = 19) and healthy controls (N = 22). Gene expression level is scaled to the sample with the lowest detected expression of a given gene.

| **Breast cancer patients** | | | | | | | | |
|---|---|---|---|---|---|---|---|---|
| **Gene** | **Number of Samples *** | | **% of Positive Samples** | **Average Expression** | **SD** | **Median** | **Expression Minimum** | **Expression Maximum** |
| | **Negative** | **Positive** | | | | | | |
| General breast cancer markers | | | | | | | | |
| *MGB1* | 12 | 7 | 37 | 119.75 | 224.41 | 0.00 | 0.00 | 718.00 |
| *HER2* | 5 | 14 | 74 | 23.87 | 27.46 | 12.6 | 0.00 | 96.50 |
| Epithelial markers | | | | | | | | |
| *CK19* | 12 | 7 | 36 | 42.40 | 127.47 | 0.00 | 0.00 | 551.60 |
| *CDH1* | 10 | 9 | 47 | 67.90 | 103.47 | 25.15 | 3.46 | 384.84 |
| Mesenchymal markers | | | | | | | | |
| *VIM* | 11 | 8 | 42 | 27.45 | 20.77 | 23.08 | 2.62 | 63.13 |
| *CDH2* | 18 | 1 | 5 | 67.90 | 103.47 | 25.15 | 3.46 | 384.84 |
| *PLS3* | 11 | 8 | 42 | 8.21 | 10.02 | 4.09 | 0.00 | 39.57 |
| Invasion-related genes | | | | | | | | |
| *CXCR4* | 8 | 11 | 58 | 9.79 | 12.24 | 5.96 | 0.00 | 48.42 |
| *uPAR* | 3 | 16 | 84 | 32.31 | 35.07 | 28.66 | 0.00 | 118.80 |
| Stem cell markers | | | | | | | | |
| *CD44* | 19 | 0 | 0 | 51.15 | 51.51 | 47.81 | 0.00 | 186.02 |
| *NANOG* | 13 | 6 | 32 | 23.13 | 22.61 | 16.95 | 0.00 | 65.46 |
| *OCT-4* | 15 | 4 | 21 | 54.18 | 64.39 | 29.05 | 0.00 | 225.23 |
| *ALDH1* | 5 | 13 | 72 | 162.38 | 176.33 | 96.98 | 0.00 | 582.56 |
| *CD133* | 16 | 3 | 16 | 63.41 | 157.71 | 19.40 | 0.00 | 706.81 |
| Cluster formation-related gene | | | | | | | | |
| *JUP* | 12 | 7 | 37 | 83.27 | 123.43 | 34.58 | 0.00 | 528.64 |
| **Healthy controls** | | | | | | | | |

| **Gene** | **Average Expression** | **SD** | **Median** | **Expression Minimum** | **Expression Maximum** |
|---|---|---|---|---|---|
| General breast cancer markers | | | | | |
| *MGB1* | 0.00 | 0.00 | 0.00 | 0.00 | 0.00 |
| *HER2* | 0.00 | 0.00 | 0.00 | 0.00 | 0.00 |
| Epithelial markers | | | | | |
| *CK19* | 0.00 | 0.00 | 0.00 | 0.00 | 0.00 |
| *CDH1* | 6.71 | 8.39 | 3.12 | 0.00 | 25.00 |
| Mesenchymal markers | | | | | |
| *VIM* | 13.00 | 9.40 | 11.4 | 0.00 | 30.7 |
| *CDH2* | 7.84 | 15.83 | 0.00 | 0.00 | 48.92 |
| *PLS3* | 2.33 | 1.84 | 2.39 | 0.00 | 4.94 |
| Invasion-related genes | | | | | |
| *CXCR4* | 0.40 | 1.40 | 0.00 | 0.00 | 4.54 |
| *uPAR* | 0.00 | 0.00 | 0.00 | 0.00 | 0.00 |
| Stem cell markers | | | | | |
| *CD44* | 75.73 | 87.61 | 32.21 | 0.00 | 272.83 |
| *NANOG* | 7.05 | 10.17 | 2.03 | 0.00 | 31.02 |
| *OCT-4* | 21.51 | 24.74 | 17.57 | 0.00 | 76.25 |
| *ALDH1* | 10.36 | 11.53 | 9.66 | 0.00 | 36.13 |
| *CD133* | 13.12 | 21.75 | 1.88 | 0.00 | 66.37 |
| Cluster formation-related gene | | | | | |
| *JUP* | 16.67 | 16.19 | 10.46 | 0.00 | 44.15 |

* Classification into positive and negative was based on the highest expression in healthy controls (applied cut-off level).

**Table S3.** Spearman correlations coefficients (ϱs) between *CD45* expression and mesenchymal markers (*VIM, CDH2* and *PLS3*) expression analysed in CTCs-enriched blood fractions of breast cancer patients. CTCs positive samples correlations are subdivided into (i) marker-positive and marker-negative samples based on the maximal cut-off level recorded in healthy controls, or (ii) into phenotypes of CTCs-EBF (epithelial, mesenchymal, epithelial-mesenchymal). Statistically significant ($p < 0.05$) results are marked in bold.

| Variable–CTCs Class | | Mesenchymal Markers | | | | | |
|---|---|---|---|---|---|---|---|
| | | *VIM* | | *CDH2* | | *PLS3* | |
| | | **ϱs** | ***p*** | **ϱs** | ***p*** | **ϱs** | ***p*** |
| CTCs-negative | | 0.89 | **<0.001** | 0.14 | 0.492 | −0.16 | 0.432 |
| CTCs-positive | All samples | 0.86 | **<0.001** | 0.45 | 0.052 | 0.08 | 0.783 |
| | Marker-positive | 0.02 | 0.955 | - | - | −0.50 | 0.207 |
| | Marker-negative | 0.78 | **0.004** | 0.41 | 0.092 | 0.45 | 0.164 |
| | Epithelial | 0.82 | **0.023** | 0.04 | 0.937 | 0.05 | 0.908 |
| | Mesenchymal | −0.31 | 0.544 | −0.20 | 0.704 | −0.60 | 0.208 |
| | Epithelial-Mesenchymal | 0.94 | **0.005** | 0.43 | 0.397 | −0.43 | 0.397 |

**Table S4.** Univariate and multivariate analysis showing the risk of death of breast cancer patients depending on the clinical variable.

| Variable | Univariate Analysis | | | Multivariate Analysis | | |
|---|---|---|---|---|---|---|
| | **HR** | **95% CI** | ***p*** | **HR** | **95% CI** | ***p*** |
| T stage (2–4 vs. 1) | 1.00 | 1–1.01 | 0.62 | 1 | 0.99–1.00 | 0.86 |
| N stage (N+ vs. N−) | 3.49 | 0.70–17.33 | 0.12 | 3.70 | 0.72–19.06 | 0.12 |
| Grading (2–3 vs. 1) | 0.97 | 0.90–1.04 | 0.38 | 0.97 | 0.90–1.05 | 0.50 |
| HR status (HR+ vs. HR−) | 1.46 | 0.18–11.86 | 0.72 | 1.47 | 0.16–13.02 | 0.73 |
| HER2 status (HER2+ vs. HER2−) | 0.86 | 0.17–4.24 | 0.85 | 0.83 | 0.14–4.94 | 0.84 |
